# Vitamin D serum levels in allergic rhinitis

**DOI:** 10.1186/1939-4551-8-S1-A67

**Published:** 2015-04-08

**Authors:** Sukran Kose, Süheyla Serin Senger, Arzu Didem Yalcin, Basak Gol Serin, Gulsun Cavdar

**Affiliations:** 1 Tepecik Training and Research Hospital, Turkey; 2 Antalya Training and Research Hospital, Turkey

## Background

Recently it has been suggested that, the worldwide increase in allergic diseases such asthma, allergic rhinitis and food allergy is associated with low serum vitamin D levels. The aim of this study was to measure serum vitamin D levels in patients with allergic rhinitis.

## Methods

Serum vitamin D (25-hydroxyvitamin D), calcium, phosphorus, alkaline phosphatase and parathyroid hormone levels were assed in 200 patients with allergic rhinitis diagnosed clinically and the results of skin prick tests for aeroallergens. Subjects with serum containing less than 20 ng/ml vitamin D were deemed deficient.

## Results

The mean vitamin D levels in the study group was found 14,7 ng/ml and 68% of patients had vitamin D deficiency.

## Conclusions

The present study showed that the majority of allergic rhinitis patients had vitamin D deficiency. Therefore measuring vitamin D serum levels could be helpful in the routine assessment of patients with allergic rhinitis.

